# Beyond the Gastrointestinal Tract: When Ingestion Turns Into Migration—A Case Series

**DOI:** 10.1155/cris/7528116

**Published:** 2026-10-30

**Authors:** Yusuf AlSaffar, Rawan Mahdi, Jaffar Alkhuzaie, Mariam Alkooheji

**Affiliations:** ^1^ Department of General Surgery Salmaniya Medical Complex–Government Hospitals Capital Governorate Manama Bahrain; ^2^ Department of Radiology Salmaniya Medical Complex–Government Hospitals Capital Governorate Manama Bahrain

**Keywords:** bowel perforation, fish bone, foreign-body ingestion, migration, retroperitoneal abscess

## Abstract

Foreign body ingestion is a common emergency presentation with a wide spectrum of symptoms. Silent ingestion of foreign bodies poses a diagnostic challenge as patients present with nonspecific symptoms for varying durations. Ingested foreign objects will pass through the body asymptomatically; however, around 1% will cause complications like bowel obstruction, perforation, and, infrequently, migration beyond the gastrointestinal (GI) system. In this case series, we present five cases of migrated ingested foreign bodies and their complications, ranging from complicated psoas muscle abscess, perianal abscess, and intraabdominal collection following small bowel perforation. A plain radiograph is the first modality used to detect the presence of a foreign body, while cross‐sectional imaging aids in assessing the nature and location of the foreign body and presence of complications. Management is often conservative with observation, while drainage by interventional radiology is advised if the collection is accessible. Surgical intervention is reserved for complex cases. High clinical suspicion, thorough history taking, and physical examination aid in reaching the diagnosis to facilitate treatment.

## 1. Introduction

Foreign body ingestion is a common emergency occurrence, with a wide spectrum of presentations that vary with age and geographic locations [1–4]. Although predominantly encountered in children, this phenomenon can also occur in adult patients [1–4]. Generally, ingested foreign bodies pass through the gastrointestinal (GI) tract asymptomatically [1–6]. However, around 1% are likely to cause complications such as bowel obstruction or hollow organ perforation [3–6]. Despite the frequency of cases, only a fraction of published reports describe the migration of swallowed foreign objects to other locations in the body. Therefore, we present five cases of migrated foreign bodies and their complications after incidental ingestion.

## 2. Case Presentation

### 2.1. Case 1

A 48‐year‐old healthy male presented to the accident and emergency (AE) department with a 10‐day history of right flank pain radiating to the groin. The patient was slightly hypotensive but otherwise vitally stable. There was mild epigastric and right hypochondriac tenderness without any peritoneal signs. Lab workup revealed a white blood count (WBC) of 20 × 10^9^/L (ref: 3.4 – 9.6 × 10^9^/L). Multiple views of abdominal radiographs were done, showcasing a radiopaque needlelike structure in the right side of the abdomen and paravertebral planes, suspicious for a foreign body (Figure 1). The patient denied any recent procedures or ingestion of bones or other objects.

**Figure 1:**
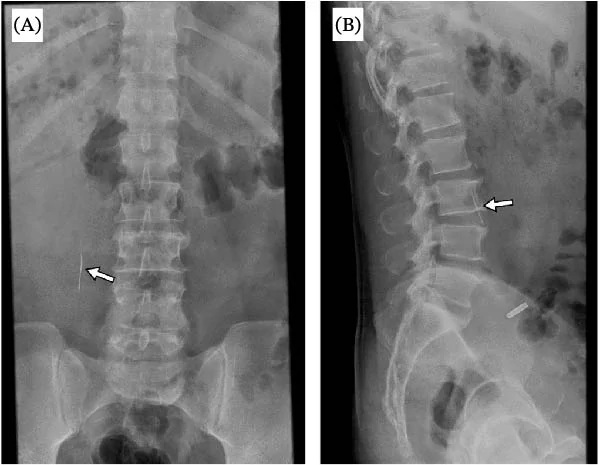
(A) Anterior–posterior plain radiograph of the abdomen and (B) lateral view of the lumbosacral spine showing a radiopaque rod‐like foreign body in the right side of the body (arrow).

Given the position of the foreign object, noncontrast computed tomography (CT) scan was initially done to rule out pathologies along the course of the urinary tract, such as ureteric calculi or calcification. A noncontrast study, followed by a contrast‐enhanced CT scan, showed suspicion of a right psoas muscle abscess and a foreign body noted within. Moreover, a 3‐cm linear object embedded in the center of a large rim‐enhancing collection measuring 2.6 × 1.8 × 7.9 cm involved the right psoas muscle (Figure 2). Significant right perinephric and retroperitoneal air pockets were also appreciated. Multiple defects were noted along the gastric pylorus and the proximal duodenum with the suspicion of intraperitoneal air pockets. Mural thickening and surrounding collections involving the hepatic flexure and proximal transverse colon, with evidence of regional inflammatory changes, were seen. The findings suggested a complicated right psoas abscess.

**Figure 2:**
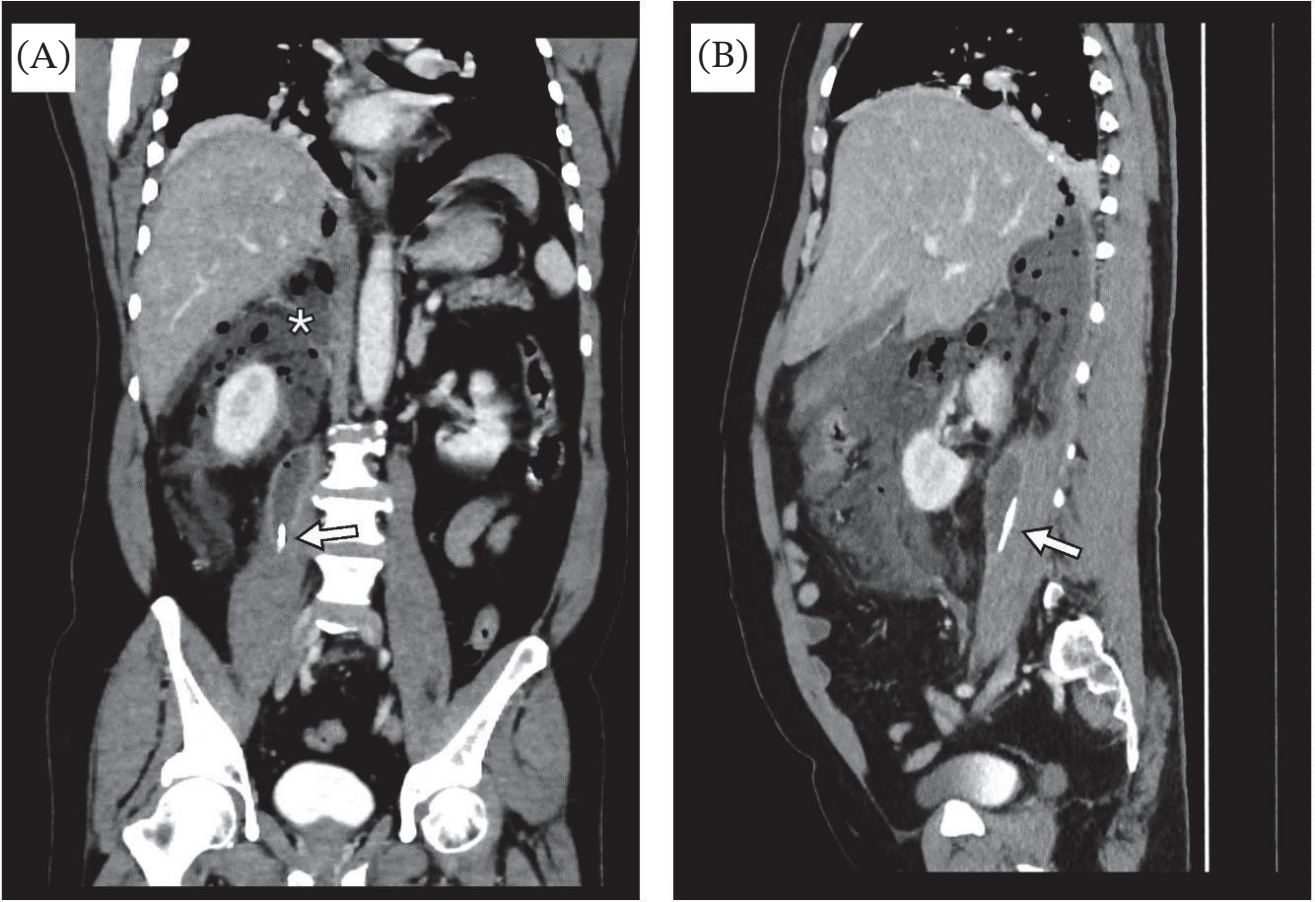
(A) Coronal cut of a contrast‐enhanced CT scan of the abdomen. Arrow showing a large rim‐enhancing collection in the right psoas muscle with a linear foreign body within. Asterisk shows a collection with air pockets extending to the right perinephric area. (B) Sagittal multiplanar reconstruction image of the same linear foreign body within the right psoas muscle collection (arrow).

A single contrast upper GI study was performed to assess GI integrity and rule out perforation as a consequence of migrated ingested foreign body; however, there was no evidence of contrast extravasation (Figure 3).

**Figure 3:**
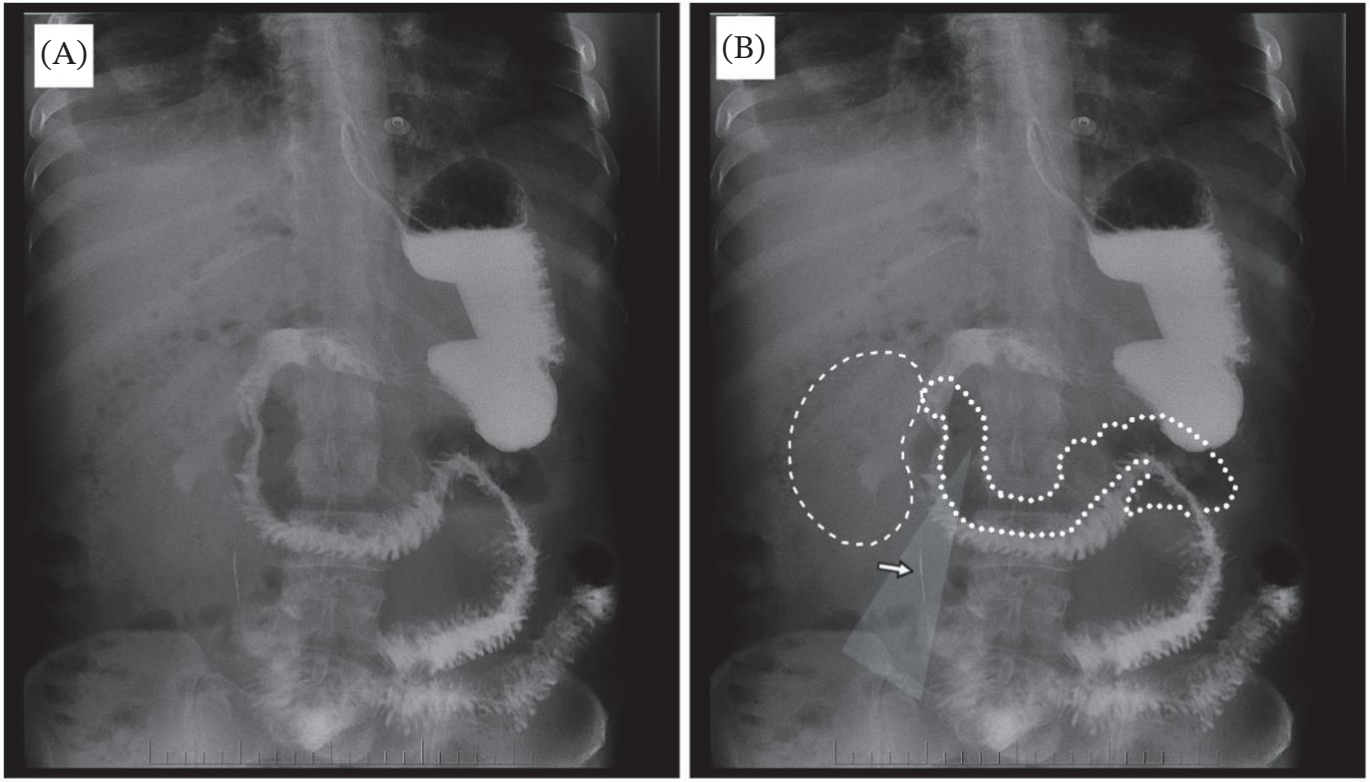
(A) Single contrast gastrografin study showing smooth flow of contrast through the esophagus, passing freely through the stomach into the small bowel loops. (B) Radiopaque rod‐like foreign body in the right side of the abdomen (arrow) at the expected location of the right psoas muscle (triangle), right kidney outline (dashed line), and transverse colon (dotted line).

The patient was clinically stable, with no peritoneal signs requiring emergency intervention. An intravenous antibiotic course was initiated, and CT‐guided percutaneous drainage of the collection was arranged. Drain output showed pus that continued for 10–15 days and gradually decreased. The antibiotic was tailored according to the microbiology analysis, which was positive for *Klebsiella pneumoniae* and extended‐spectrum beta‐lactamases *Escherichia coli* strains. After 23 days on Meropenem, the patient improved without surgical intervention. The patient was discharged home; however, lost follow‐up as he returned to his home country.

### 2.2. Case 2

A healthy 42‐year‐old gentleman presented to AE with a 1‐week history of painful perianal swelling. He denied any history of fever or discharge from the site of swelling. On examination, no obvious swelling could be appreciated at the anal verge; however, there was severe tenderness at the 9 o’clock position as well as minimal pus discharge. No signs of anal trauma or sexual abuse were appreciated. The lab workup revealed a WBC of 13 × 10^9^/L. The patient underwent anorectal examination under anesthesia with the incision and drainage of the perianal abscess. Intraoperative findings showed a perianal abscess cavity 2 cm away from the anal verge extending to the ischiorectal space. Incidentally, the operating surgeon felt a pointed, sharp object, which turned out to be a fish bone extracted from the abscess cavity (Figure 4). No obvious breach of rectal integrity was detected. The wound was packed, and the patient was admitted for observation and antibiotics. After surgery, the patient admitted to eating seafood recently; however, he did not recall swallowing any fish bone. The patient was discharged without any complications.

**Figure 4:**
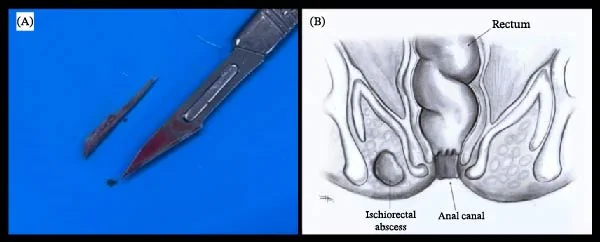
(A) Fish bone, measuring 2–3 cm, incidentally found within the ischiorectal abscess and (B) illustration of the ischiorectal abscess.

### 2.3. Case 3

A 26‐year‐old healthy male was brought by the ambulance to the AE with severe anal pain and limited mobility for a couple of days. A hard structure was felt by the patient at the anal verge; however, he denied any history of foreign‐body insertion or ingestion. Apart from local tenderness on digital rectal examination, no foreign body was appreciated initially in the anorectal canal. A CT scan was performed, which featured a 3‐cm hyperdense linear structure situated within the rectum with no other abnormalities (Figure 5). Anoscopy was then done for the patient, and a wooden object was visualized (Figure 6). Upon further questioning, the patient admitted to eating grilled corn on a wooden skewer the day before, which could be the source of the ingested wood shard. Anoscope assessment in the operating room showed a wooden foreign body lying transversely below the dentate line, puncturing the anal canal wall from 3 to 9 o’clock. The foreign body was removed safely, and the patient recovered without complications.

**Figure 5:**
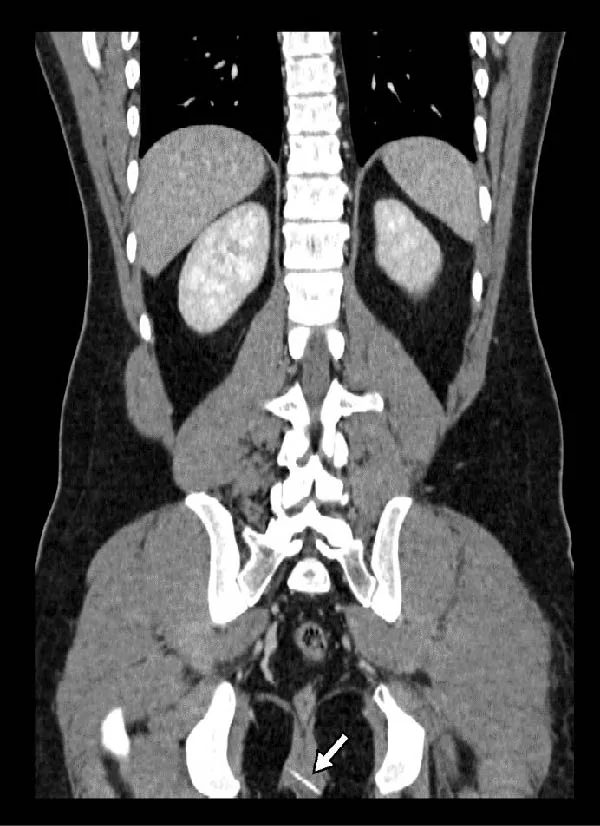
Coronal cut of a contrast‐enhanced CT scan of the abdomen. Arrow shows a 3‐cm‐linear hyperdense structure lying transversely within the rectum.

**Figure 6:**
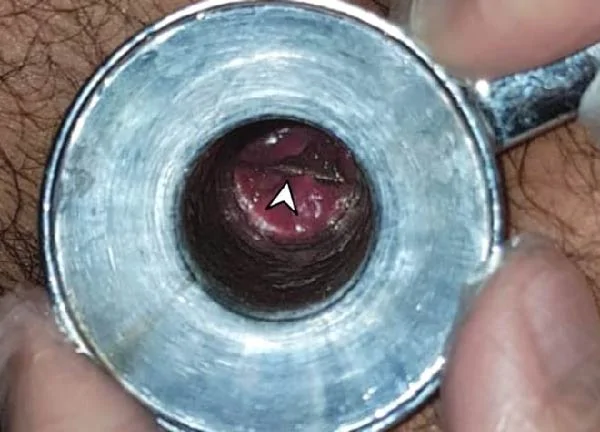
Anoscopy examination showing the wooden foreign body lying transversely within the rectum (arrowhead).

### 2.4. Case 4

A 56‐year‐old male with hypertension, ischemic heart disease, stroke, and a single kidney presented to the AE with a 1‐week history of progressive diffuse abdominal pain, aggravated by movement. Additionally, he mentioned chronic constipation and a loss of appetite. Investigations showed a WBC of 10.13 × 10^9^/L, and fecal loading was noted on plain radiographs. Suspicion of an occult perforated viscous or perforated appendix was raised due to the presence of localized peritoneal signs.

A CT scan revealed a linear hyperdense foreign body consistent with a fish bone seen within an inflammatory mass‐like region involving thickened small bowel and the adjacent mesentery (Figure 7). Extraluminal gas was noted with a possible tract of penetration through the proximal ileal loops. After further inquiry, the patient recalled eating fish within a week of presentation. Exploratory laparotomy and small bowel resection were performed.

**Figure 7:**
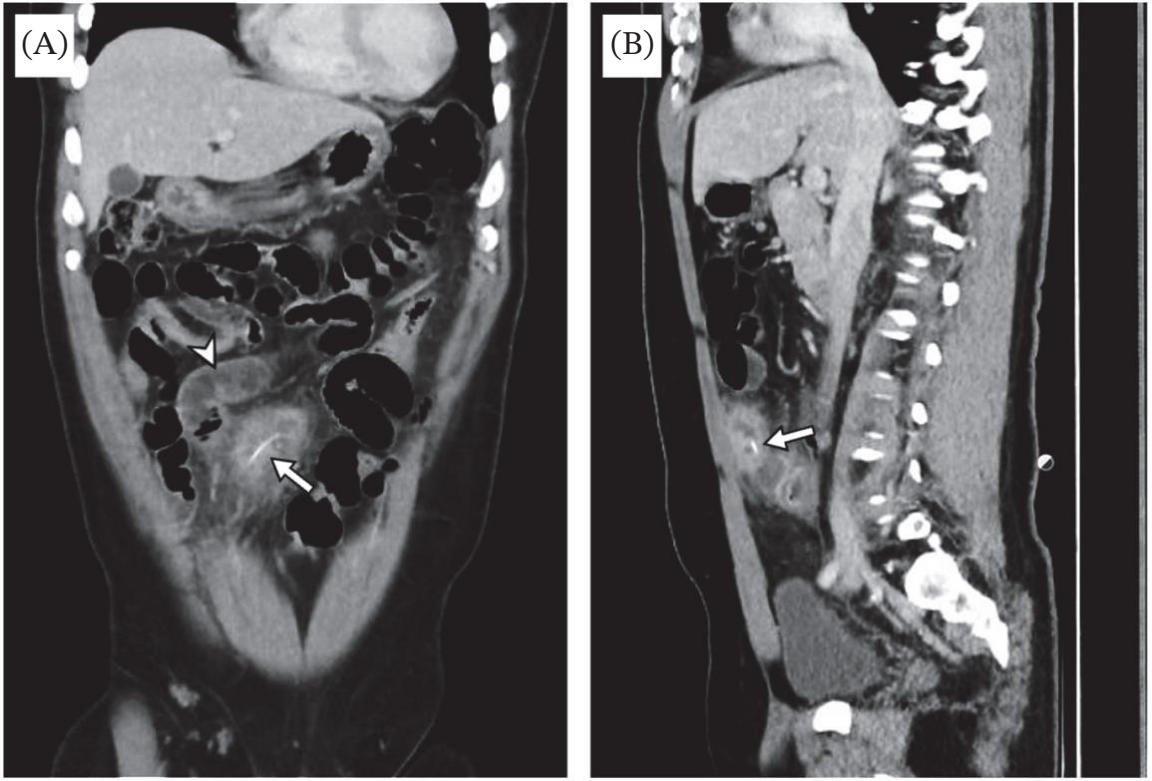
(A) Coronal cut of a contrast‐enhanced CT scan of the abdomen. Arrow showing a linear foreign body within an inflammatory mass‐like region of the mesentery, arrowhead demonstrating thickened bowel loops. (B) Sagittal cuts show the same linear foreign body (arrow).

Small bowel perforation was noted at a distance of 250 cm proximal to the ileocecal valve, with an inflammatory mass involving adherent omentum and an unhealthy bowel. The foreign body in question could not be isolated from the inflammatory mass intraoperatively; therefore, the unhealthy bowel segment was resected, and side‐to‐side anastomosis was done. The patient remained on antibiotics throughout his hospital stay and was discharged without complication. Subsequently, histological assessment confirmed a fish bone embedded within an organized omental abscess.

### 2.5. Case 5

A 76‐year‐old gentleman presented with acute left loin pain for 2 days duration. No nausea or vomiting were reported. The patient was vitally stable, with mild left hemiabdomen tenderness extending to the suprapubic region. His lab results and plain radiographs were unremarkable. A noncontrast abdominal CT scan was arranged to exclude renal pathologies in view of the presenting complaint. A linear hyperdense structure measuring 3 cm was embedded along the lateral aspect of the spleen associated with surrounding fat stranding. A small hypodense subscapular hematoma/collection was seen on a follow‐up contrast‐enhanced study. This raised the possibility of an extraluminal‐migrated ingested foreign body (Figure 8). After discussing the findings with the patient, he remembered eating fish 3 days prior to the onset of symptoms; however, he did not sense any swallowed fish bones. The patient was observed, managed conservatively, and later discharged in stable condition.

**Figure 8:**
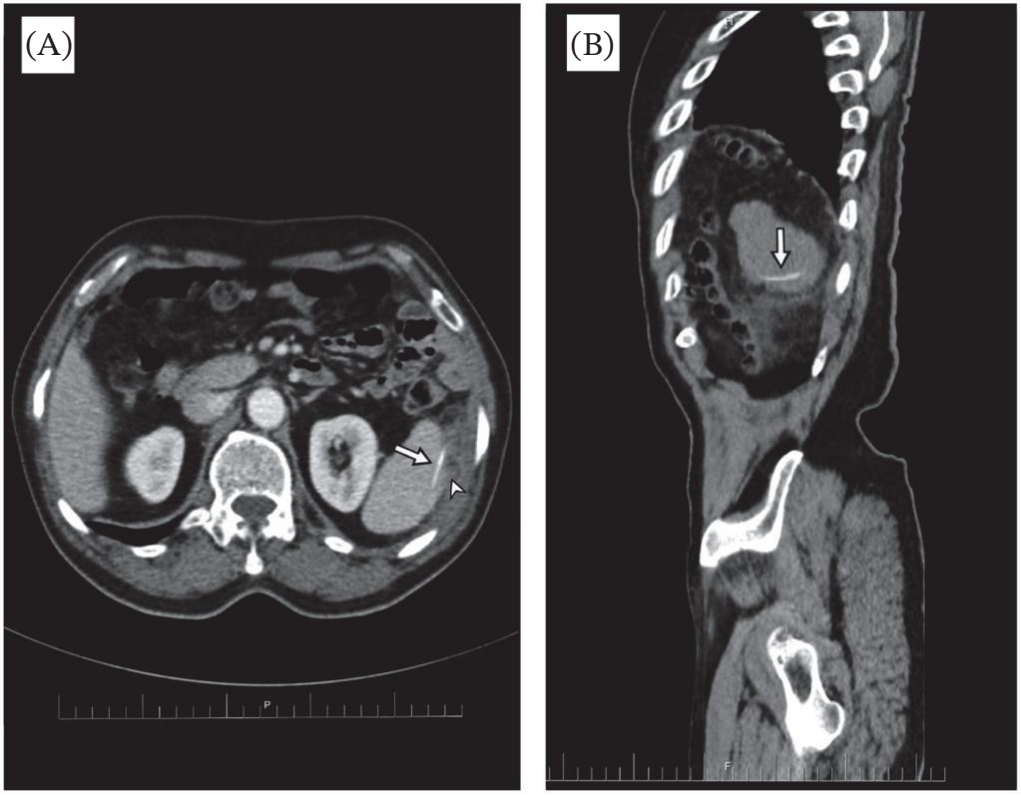
(A) Axial cut of a contrast‐enhanced CT scan of the abdomen. (Arrow) showing a linear foreign body within the spleen. Arrow head indicates a hypodense subcapsular region suggestive of a small hematoma/collection. (B) Sagittal cuts show the same linear foreign body (arrow).

## 3. Discussion

Foreign body ingestion is a common emergency presentation [1–4]. Underlying psychiatric illnesses, alcohol intoxication, and senile conditions might play a role in the history of swallowing foreign objects [2–4, 7]. Moreover, most accidental ingestions are through dietary sources, such as fish bones (33%) and chicken bones (12.5%), or sharp objects held in the mouth, like toothpicks (27.3%) and needles (9.1%) [2, 4, 8, 9].

Patients often seek medical attention knowing they have swallowed something by accident [6]. Silent ingestions, where the patients are unaware of swallowing a foreign body, pose a challenge. These cases might present with delayed, nonspecific symptoms like abdominal pain, nausea, diarrhea, or per‐rectal bleed [1, 4, 6, 10]. Some presentations may be obscured by other obvious diagnoses, masking the original pathology. For example, Case 2 (perianal abscess) was a consequence of a migrated fish bone as opposed to a perianal fissure or fistula. Similarly, Case 5 was perceived as a renal pathology with loin pain, which surprisingly turned out to be a fish bone migrated to the spleen.

Obtaining a pathognomonic history in such cases can be challenging, as patients cannot recall ingesting a foreign body to begin with [10–12]. Four of the cases detailed were medically free, healthy adults who presented to the emergency department with nonspecific complaints for different durations. Most of them denied a history of foreign‐body insertion, trauma, or ingestion at the time of presentation, and the foreign bodies were only discovered after further workup was conducted. Patients with existing comorbidities require additional caution in diagnosis and management options depending on their medical fitness. A prime example is Case 4, with features of chronic constipation versus bowel pathologies with peritoneal signs.

A plain radiograph is the initial modality to detect the presence of a foreign body [1, 4, 6]. Batteries, pins, and coins will present as radiopaque structures, while fish and chicken bones, wood, and plastic can be missed as they often appear radiolucent [6]. Radiographs lose their value depending on the presentation; hence, Case 2 (perianal abscess) and Case 3 (rectal pain) did not warrant any initial imaging. A contrast‐enhanced CT scan is more superior in properly assessing the nature and location of the foreign bodies and to rule out any concerning complications [1, 4, 6]. The first case showcased a foreign body incidentally discovered on an abdominal radiograph for vague abdominal pain. Cross‐sectional imaging later revealed a complex iliopsoas abscess. Cases 4 and 5 had a similar outcome in that the foreign body was found within an intraabdominal collection and solid organ, respectively, after breaching a bowel segment.

Symptoms vary in presentation, duration, and severity depending on multiple factors. Approximately 80%–95% of ingested foreign bodies will travel through the GI tract asymptomatically until they are expelled within a 1‐week period [1, 3–5, 7]. Most patients become symptomatic within days to weeks from ingestion, while exceptional cases exhibit a latency period [4, 13, 14]. Reported cases in the literature describe foreign bodies lasting as long as 9 months, some even for 8 years [5, 7]. A small percentage of foreign bodies will cause complications such as bleeding, abscess formation, bowel obstruction, or perforation, especially with elongated and sharp objects [4–13].

Extraluminal migration of foreign bodies is rare but not completely unheard of [2]. Several sources in the literature have theorized about the possible routes of migration of ingested foreign bodies. The stomach and duodenum appear to be favorable locations for foreign‐body complications [4, 5, 8, 11, 12]. The duodenum’s retroperitoneal position and angulation render the short, maze‐like segments difficult for a long object to traverse [4, 5, 11]. For example, in Case 1, the most likely point of breach is between the second and third parts of the duodenum, which correlates with CT scan findings affecting the transpyloric plane and ending with the foreign object embedded in the retroperitoneal psoas muscle (Figure 9). This explains the delayed presentation of the patient and the absence of peritoneal signs. The ileum and ileocecal valve are yet other possible locations for perforation (Case 4) due to their narrow lumen, which may obstruct the passage of a foreign body [1, 4, 9, 12, 15]. In rare cases, perforation can occur at the colonic flexures due to their fixed, narrow positions and sharp angles [15, 16]. For instance, Case 5 showcased a foreign object that likely penetrated the splenic flexure and migrated to the spleen (Figure 10). Long and sharp objects may gradually penetrate the thick muscular wall of the GI system, allowing the perforation to seal spontaneously, masking the symptoms until infection and abscess formation ensue along the path of migration [12, 14, 15].

**Figure 9:**
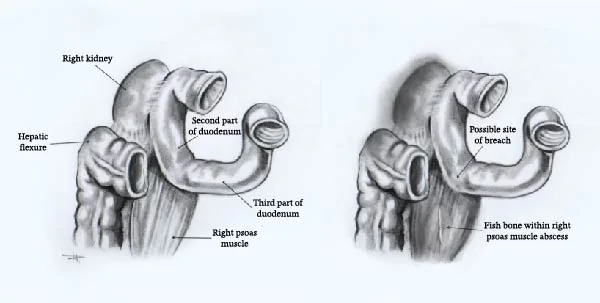
Illustration of the possible point of breach between the second and third parts of the duodenum, leading to the foreign body migrating to the retroperitoneal psoas muscle as in Case 1.

**Figure 10:**
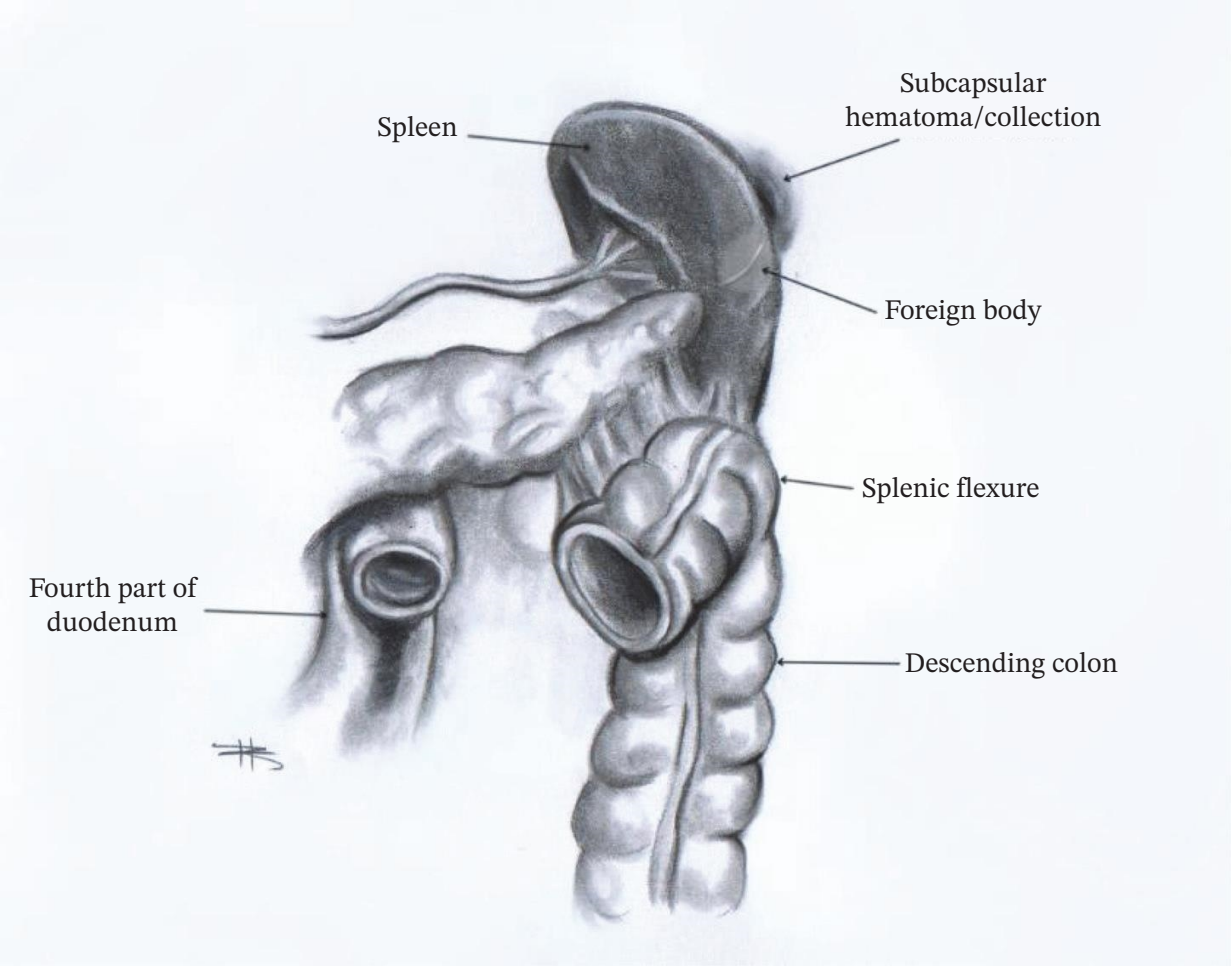
Illustrates the fixed position and sharp angulation of the splenic flexure, a likely point of penetration for the foreign body migrating to the spleen as in Case 5.

In acute presentations, migrated extraluminal foreign bodies can cause variable complications such as perforation with peritonitis, obstruction, fistulas, and abscess formation [1, 3, 12, 15]. Patients with silent ingestions often present to the AE with secondary features after the inflammation and infectious process have settled in the affected organs, such as in Case 5 (spleen). Extremely unusual cases of migrated foreign bodies have also been reported involving the vena cava and the spinal cord [2, 17, 18].

Manifestations of symptoms may be attributed to the shape, size, and type of material ingested. Bacterial infection may further propagate the inflammatory cascade initiated by the foreign body. Notable strains cultured include Streptococcus, *Escherichia coli*, and *Klebsiella pneumonia*, respectively (Case 1) [8].

The pathway and final destination of a migrated foreign body throughout the GI tract dictate management options [1]. The clinical picture, severity of symptoms, and the traversed planes, namely, intraperitoneal or retroperitoneal spaces, also play a role in treatment [1]. The majority of foreign objects will be naturally expelled from the body and are therefore managed conservatively [12, 15]. Retained foreign bodies can be surgically removed when they are symptomatic. Otherwise, they can be kept for observation if surgery is considered invasive, clinically high risk, or carries a higher rate of complications compared to benefits (Case 1 and 5) [17, 18]. Complicated cases with abscess formation require drainage procedures and antibiotic coverage [8, 12, 15]. Therapeutic options depend on the patient’s clinical stability and accessibility to an inflammatory collection, where patients can be deferred for a minimally invasive drain insertion under radiological guidance [8, 14, 15]. Conservative management offers favorable outcomes; however, it is limited by its inability to extract the offending object. This, in theory, can harbor a potential source of infection and recurrence in the future. Endoscopic procedures can be helpful in early presentations with knowledge of accidental ingestion, as they aid in retrieval of the foreign bodies from the proximal alimentary tract prior to development of complications [12, 14, 15]. Laparoscopic or open exploration of the abdomen should be reserved for patients who are unstable or have signs of frank perforation [8, 12, 14, 15]. These procedures provide rapid access, immediate decontamination, and the added benefit of extraction of the toxic foreign body, which results in a shorter hospital course [19].

## 4. Conclusion

Migration beyond the GI system is one of the determinant consequences of ingested foreign bodies. Diagnosing silent ingestions is challenging, and this case series highlights the importance of clinical history, examination, understanding of surgical anatomy, and approach as key elements in managing patients optimally. Prompt detection and evaluation of the offending material improve the overall hospital course. Management options should be tailored by each case, and the hallmark of treatment should focus on management of infection and extraction of foreign material if feasible with the least amount of damage to the affected organ and traversed path. Physicians should possess a high clinical suspicion and be thorough in their investigations. Effective communication between the involved specialties, such as emergency, surgical, and radiological, is imperative for efficient patient care.

## Funding

No funding was received for this manuscript.

## Ethics Statement

The Research & Research Ethics Committee for Government Hospitals at Salmaniya Medical Complex have issued an approval for the case series (Reference Number GH‐REC‐10‐2026‐074857).

## Consent

The authors confirm that informed consent has been obtained from the patients for this information to be published in this case series and accompanying images.

## Conflicts of Interest

The authors declare no conflicts of interest.

## Data Availability

The data that support the findings of this study are available from the corresponding author upon reasonable request.
